# Vertical Stratification in Composition, Metabolic Potential and Co‐Occurrence Networks of Archaeal Communities in Mangrove Sediments, Zhanjiang, China

**DOI:** 10.1002/ece3.74279

**Published:** 2026-09-08

**Authors:** Yunru Li, Songze Chen, Qing Li, Yue Sun, Dan Sun, Mingzhong Liang, Wei Zhang, Jianqing Chen, Jing Sun, Bin Gong, Jingjing Song, Ying Liu, Rongping Bu

**Affiliations:** ^1^ Pinglu Canal and Beibu Gulf Coastal Ecosystem Observation and Research Station of Guangxi, Guangxi Key Laboratory of Marine Environmental Disaster Processes and Ecological Protection Technology, College of Marine Sciences Beibu Gulf University Qinzhou China; ^2^ Shenzhen Ecological and Environmental Monitoring Center of Guangdong Province Shenzhen China; ^3^ Fangchenggang Vocational and Technical College Fangchenggang China

**Keywords:** archaeal communities, community assembly, environmental driving factors, functional prediction, mangrove wetlands, network interactions, vertical distribution

## Abstract

Archaea, a pivotal domain in the global biosphere, serve as indispensable mediators in biogeochemical turnover of mangrove wetlands. Nonetheless, high‐resolution vertical changes of archaeal communities, function, co‐occurrence patterns, and assembly mechanisms as well as their driving factors are poorly described in the mangrove sediments. Herein, through high‐throughput analysis of archaeal communities across seven depth intervals (0–90 cm) within 8 sediment cores collected in the Zhanjiang mangrove wetlands, we provide the vertical profiles of archaeal community composition and function, co‐occurrence networks, and assembly mechanisms along the sediment depth. Our results show that archaeal abundance declined with sediment depths, while higher archaeal diversity was observed in the mangrove deep sediments. Thermoplasmatota was more abundant in surface mangrove sediments, whereas Thermoproteota (Bathyarchaeia) was remarkably enriched in deep sediments. Metabolically, surface archaeal communities are characterized by carbohydrate, amino acid, cofactor and vitamin metabolism, whereas glycan biosynthesis, xenobiotics biodegradation, and nucleotide metabolism serve as the predominant functions in deep archaeal communities. The surface network displayed greater complexity and stability than the deep counterpart, with Thermoproteota functioning as keystone taxa across all three networks. Further, stochastic processes govern archaeal community assembly in all sediment layers. Finally, sediment depth, temperature, moisture, TP, clay content, and salinity were identified as key drivers shaping archaeal communities. These findings expand our knowledge regarding the vertical distribution characteristics and key driving factors of mangrove archaea, while also underscoring their ecological importance in deep mangrove sediment habitats.

## Graphical abstract

We characterized the vertical profiles of archaeal community composition, function, co‐occurrence networks, and assembly mechanisms in mangrove sediments across six depth intervals. Deep sediments hosted higher archaeal diversity, with distinct taxonomic and functional shifts, and more stable co‐occurrence networks compared to surface layers. Stochastic processes (dispersal limitation) primarily governed community assembly, with sediment depth, TN, TP, and δ^15^N acting as key environmental drivers.

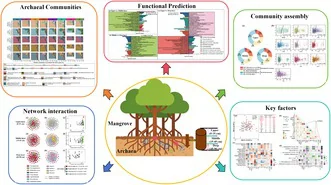

## 1 Introduction

Mangrove wetlands represent critical “blue carbon ecosystems”, predominantly located within tropical and subtropical intertidal zones globally (Alongi 2014; Bunting et al. 2022; Contessa et al. 2023; Donato et al. 2011; Laux et al. 2024). Spatial heterogeneity in mangrove productivity, tidal fluctuation, and nutrient availability, in conjunction with other influential factors collectively shape biocomplexity inherent to mangrove ecosystems (Feller et al. 2010). A number of investigations have consistently reported that mangrove surface and deep sediments exhibit distinct difference in various environmental conditions (Ding et al. 2025; Luo et al. 2021; Qi et al. 2025). For instance, mangrove surface sediments (0–20 cm) were characterized by higher oxygen levels, elevated labile organic matter content, lower salinity (2.2‰–8.9‰), and pH (5.5–6.5). In contrast, deep sediments (> 30 cm) exhibited anoxia, recalcitrant organic matter dominance, predominantly hypersaline conditions (25–35 PSU), and pH (6.8–7.5) (Kristensen et al. 2017; Tang et al. 2023). These singular habitat conditions foster highly diverse and complex microbial assemblages, that are critical to regulating nutrient biogeochemical turnover (Bhattacharyya et al. 2015; Lin et al. 2019; Yu et al. 2023; Zhang, Chen, Sun, et al. 2021; Zhang et al. 2019; Zhang, Pan, et al. 2021).

Archaea, serving as pivotal constituents of the microbial community, were ubiquitously distributed across mangrove wetlands (Bhattacharyya et al. 2015; Pan et al. 2019; Zhang, Pan, et al. 2021; Zou et al. 2025). Archaea constitute 20.8%–41.3% of the prokaryotic community (Luis et al. 2019), coupled with archaeal abundance ranging from 10^7^–10^10^ copies g^−1^ dw in mangrove ecosystems (Zhou et al. 2017). Owing to vertical variations in sediment physicochemical properties, archaeal communities exhibit distinct vertical stratification within mangrove wetlands. Multiple previous studies have demonstrated that Thaumarchaeota, Woesearchaeota, and Euryarchaeota constituted the major archaeal groups in surface layers of mangrove sediments, whereas Crenarchaeota, Thaumarchaeota, Asgardarchaeota, and Euryarchaeota were enriched in deep layers (Mendes et al. 2012; Ng et al. 2025; Pan et al. 2019; Zhang et al. 2018; Zhang, Pan, et al. 2021). Meanwhile, surface mangrove sediment archaeal assemblages were mainly involved in aerobic respiration, iron reduction, and denitrification, whereas those in deep sediments were dominated by sulfate reduction and methanogenesis (Kristensen et al. 2017). As revealed in multiple preceding studies, temperature, salinity, pH, sediment depth, and total organic carbon (TOC) exert dominant regulatory effects on archaeal communities within mangrove wetlands (Fiard et al. 2022; Zhang, Pan, et al. 2021). To date, our comprehension of archaeal community structure and function in mangrove ecosystems remains largely confined to surface sediments, with limited insights into the archaeal communities inhabiting deeper layers.

Microbial interactions constitute a core mechanism underpinning the high productivity of mangroves, the stability of carbon‐nitrogen‐sulfur biogeochemical turnover, and habitat resilience (Abatenh et al. 2018; Palit et al. 2022; Qian et al. 2023). Microbial co‐occurrence network approaches have proven to be effective tools for revealing these complex inter‐microbial relationships (Hou et al. 2021; Hu et al. 2021). While prior investigations have delineated the network interaction patterns of archaeal communities across mangrove wetlands (Chen and Wen 2021; Zhang, Pan, et al. 2021), critical knowledge gaps remain concerning how network interactions and keystone taxa differ among archaeal communities in surface, middle, and deep mangrove sediment layers.

Microbial community assembly, driven by the joint action of deterministic and stochastic processes, serves as a pivotal regulator of microbial diversity, spatial distribution, and succession dynamics (Chen et al. 2019; Hanson et al. 2012; Stegen et al. 2012; Zhou et al. 2017). Multiple quantitative models, including the beta nearest‐taxon index (βNTI) (Stegen et al. 2012) and phylogenetic bin‐based null model analysis (iCAMP) (Ning et al. 2020), were used to quantify the specific impacts of stochastic and deterministic processes on microbial community assembly. Typically, both deterministic and stochastic processes concurrently modulate microbial community assembly, with the dominant process varying across different studies (He et al. 2021; Zhang et al. 2023). Nevertheless, how archaeal community assembly mechanisms differ across mangrove sediment layers remains poorly understood.

Elucidating the vertical stratification in the structure and functional potential of archaeal communities across mangrove sediment profiles and identifying their key environmental drivers is essential for revealing the ecological roles of archaea in deep mangrove sediments. To address this objective, 8 sediment cores (0–90 cm) were collected from the eight distinct sampling sites across the Zhanjiang mangrove wetlands. The present research was framed around three principal objectives: (i) to compare the compositional characteristics and metabolic potential of archaeal communities across upper, middle, and deep mangrove sediment layers, (ii) to delineate differences in archaeal co‐occurrence network topology and community assembly mechanisms across surface, middle, and deep sediment layers, and (iii) to determine the principal environmental drivers governing the structure of the mangrove archaeal community. Deciphering how mangrove archaeal communities respond to sediment depth provides a theoretical framework for quantitatively predicting their functional roles in biogeochemical cycling within deep mangrove habitats.

## 2 Materials and Methods

### 2.1 Sample Collection and Physicochemical Analysis

**FIGURE 1:**
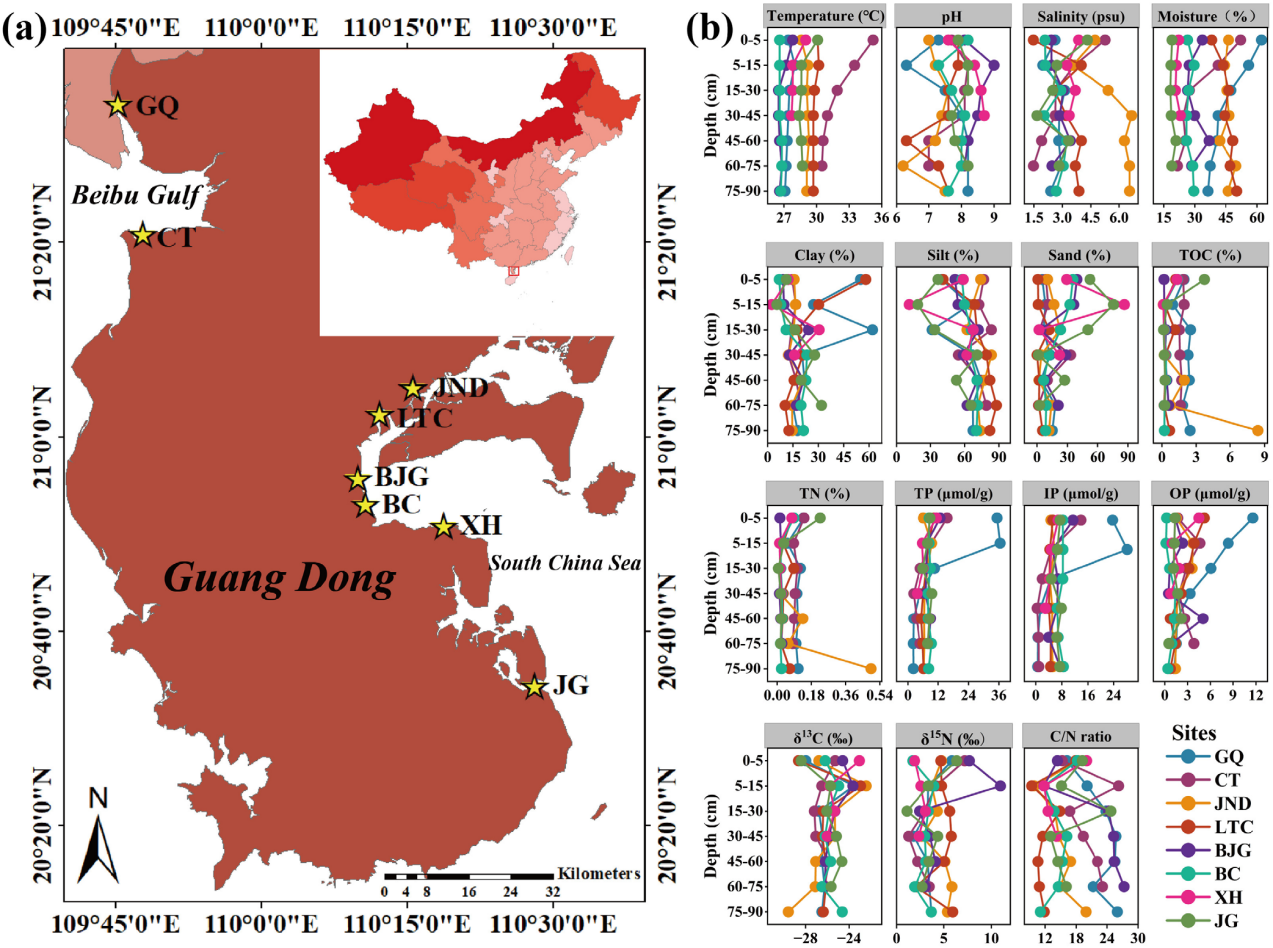
(a) Location of study area and sampling sites (shown as yellow asterisks) from the eight different locations in Zhanjiang mangrove wetlands of Guangdong, China. Gaoqiao Mangrove Nature Reserve (GQ), Caotan Town (CT), Jinniu Island (JND), Lingtou Village (LTC), Beijia Gang (BJG), Beicun Village (BC), Xiahu Village (XH), and Jingou Village (JG). (b) The vertical variations of temperature, pH, salinity, moisture, clay, silt, sand, total organic carbon (TOC), total nitrogen (TN), total phosphorus (TP), inorganic phosphorus (IP), organic phosphorus (OP), δ^13^C, δ^15^N, and C/N ratio in the core sediments (0–90 cm).

Eight sediment cores (0–90 cm) were obtained from eight distinct sampling sites across the Zhanjiang mangrove wetlands from Guangdong, China, in September 2023. These sampling locations mainly included Gaoqiao Mangrove Nature Reserve (GQ), Caotan Town (CT), Jinniu Island (JND), Lingtou Village (LTC), Beijia Gang (BJG), Beicun Village (BC), Xiahu Village (XH) and Jingou Village (JG). The sediment cores were divided into secven depths (i.e., 0–5, 5–15, 15–30, 30–45, 45–60, 60–75, and 75–90 cm), resulting in a total of 50 samples. Nevertheless, the sediment core sampling depths at four locations (CT, BJG, XH, and JG) failed to reach 90 cm owing to the presence of bedrock. Specifically, CT, BJG, and JG lacked the 75–90 cm layer, while the XH site missed all samples from the 45–90 cm depth range. Specifically, the 75–90 cm layer was absent at CT, BJG and JG, whereas all samples spanning 45–90 cm were missing at the XH site. Following the stratification strategy reported by Qian et al. (Qian et al. 2023), we grouped all seven depths into three layers: surface (0–15 cm), middle (15–45 cm), and deep (45–90 cm). The spatial location of the mangrove sampling sites was depicted in Figure 1a. Subsequently, the sediments were split into two aliquots: one maintained at −80°C for molecular biological analysis, and the residual sediments were kept at −20°C for measures of environmental parameters after being transported to the laboratory immediately. Determination of fifteen environmental factors, including temperature, salinity, pH, moisture content, silt, sand, clay, total organic carbon (TOC), total nitrogen (TN), total phosphorus (TP), inorganic phosphorus (IP), and organic phosphorus (OP), δ^13^C, and δ^15^N, was performed following the previous methods (Liu et al. 2024).

### 2.2 Real‐Time Quantitative PCR

Genomic DNA was extracted and quantified according to the protocols described by Liu et al. (2024). Quantification of the archaeal absolute abundance of sediment samples was conducted on a Rocgene Archimed X6 Real‐Time PCR Detection System. The archaeal 16S rRNA gene was achieved with the primer set Uni519F (5´‐CAGCMGCCGCGGTAA‐3′) and Arch908R (5´‐CCCGCCAATTCCTTTAAGTT‐3′) (Wang et al. 2019). The PCR amplification system was composed of 10 ng DNA, primers (10 μM), 5 μL SYBR Premix Ex Taq, and ddH_2_O. Triplicate reactions were performed for each sample. The qPCR amplification program followed the previous literature (Liu et al. 2024). A standard curve (Ct value vs. gene copy number) was created using 10‐fold serial plasmid dilutions of DNA carrying the archaeal 16S rRNA gene amplicon fragment, and the amplification efficiencies varied from 90%–110%, with an R^2^ value greater than 0.99.

### 2.3 PCR Amplification of Archaeal 16S rRNA Gene

The V4–V5 region of the archaeal 16S rRNA gene was targeted for amplification from sediment DNA via barcoded primers 515F (5´‐GTGYCAGCMGCCGCGGTAA‐3′) and 926R (5´‐CCGYCAATTYMTTTRAGTTT‐3′) (Fadeev et al. 2021). Each reaction system was composed of Premix Taq, primer (10 μM), template DNA, and DNase‐free ddH_2_O. PCR amplification was conducted on a GeneExplorer thermocycler (BIOER) with the cycling protocol (Liu et al. 2024). Both negative and positive controls were included in each amplification batch to ensure experimental validity. After purification, all PCR amplicons were assessed via a Qubit 4 Fluorometer (Thermo Fisher Scientific, USA).

### 2.4 High‐Throughput of Archaeal 16S rRNA Gene and Data Processing

Then, these PCR products were pooled at equimolar concentrations and sequenced on the MGISEQ‐2000RS with PE300 mode. Amplicon data processing was carried out in accordance with the standard workflow established by the Fuhrman Lab. After quality control of raw reads (Martin 2011), the quality‐filtered 16S rDNA amplicon sequences were processed for truncation, denoising, and chimera filtering using DADA2 algorithms. Taxonomic annotation of the 16S rRNA gene amplicon sequence variants (ASVs) was subsequently implemented with the classify‐sklearn classifier in QIIME 2, based on alignment against the SILVA 138.1 reference database (Bolyen et al. 2019). These procedures yielded 6045–358,619 sequences across 50 distinct samples, and all samples underwent rarefaction to ensure a consistent minimum sequencing depth of 5110 sequences per sample.

### 2.5 Statistical Analysis

The location of mangrove sampling sites was mapped with ArcGIS 10.8.1. A Venn diagram analysis, implemented via the online tool (https://www.omicstudio.cn/tool/6), quantified the relative proportions of unique and shared ASVs across surface and deep sediment horizons. The construction of the archaeal community phylogenetic tree was accomplished via MEGA v11 (Kumar et al. 2016), and the bubble charts were acquired via TBtools (Chen et al. 2020). The non‐metric multidimensional scaling (NMDS) and analysis of similarity (ANOSIM) were conducted to determine the differences of archaeal community via the PRIMER v7 software (Clarke and Gorley 2015). Linear discriminant analysis Effect Size (LEfSe) was utilized to screen potential biomarkers within the archaeal community at different taxonomic ranks, with an LDA value of 4.1 and a significance level of *p* < 0.05 (https://www.omicstudio.cn/tool). Prediction of Kyoto Encyclopedia of Genes and Genomes (KEGG) metabolic pathways was conducted using PICRUSt2 (version 2.6.3) (Douglas et al. 2020; Wright and Langille 2025). Differential metabolic pathways across surface, middle, and deep sediment layers were screened in *R* with thresholds of | log_2_(fold change) | ≥ 1 and false discovery rate (FDR) < 0.05. Redundancy analysis (RDA) was implemented by CANOCO 5 software to quantify the effects of environmental variables on the archaeal community structure. Analysis of Spearman correlations within the R software framework was utilized to generate heatmaps that visualize the interrelationships between the archaeal consortia and diverse environmental variables.

### 2.6 Co‐Occurring Network Analysis

To characterize the ecological interactions among archaeal communities in surface and deep sediment layers of the Zhanjiang mangrove wetlands, co‐occurrence network analyses were carried out. These analyses adhered to the online pipeline (MENA; http://ieg4.rccc.ou.edu/mena) (Zhou et al. 2010, 2011). Network hubs, module hubs, and connectors were designated as keystone, which are critical to shaping archaeal community structure and potential functional profiles (Deng et al. 2012). The network was visualized via Gephi v0.9.2 (Bastian et al. 2009).

### 2.7 Assembly Patterns of Archaeal Communities

To quantify the relative contributions of deterministic (homogeneous and heterogeneous selection) and stochastic processes (dispersal limitation, homogenizing dispersal, drift, et al.) to archaeal community assembly across sediment depth gradients, all samples were reclassified into three depth groups: surface (0–15 cm), middle (15–45 cm), and deep (45–90 cm). The iCAMP analytical framework was utilized through the galaxy‐hosted pipeline (Ning et al. 2020). The βNTI values of archaeal communities were calculated via an online pipeline (https://www.bioincloud.tech/standalone‐task‐ui/dynamic_Rcbray_test0013). To further elucidate the relationships between βNTI values of archaeal community and geochemical parameters, Pearson's *r* coefficients and *p* values were determined by Origin 2025.

## 3 Results

### 3.1 Vertical Variation of Physicochemical Characteristics in Mangrove Sediment

Comprehensive analyses of fifteen physicochemical parameters were performed on 50 sediment samples (0–90 cm depth) from Zhanjiang mangrove wetlands (Figure 1b). Overall, the temperature, pH, salinity, and moisture of these samples exhibited ranges of 26.5°C–35.2°C, 6.2–9.0, 1.5–6.7 psu, and 18.2%–62.2%, respectively. Sediment particle size analysis showed that the proportions of silt, sand, and clay across all samples varied between 11.1%–87.8%, 0.1%–86.5%, and 2.4%–62.0%, respectively, with silt constituting the dominant particle fraction. For nutrient properties, the total organic carbon (TOC) and total nitrogen (TN) concentrations of the samples varied within the intervals of 0.1%–8.4% and 0.0066%–0.4938%, respectively. Concentrations of total phosphorus (TP), inorganic phosphorus (IP), and organic phosphorus (OP) ranged from 2.1–36.5 μmol/g, 0.4–28.1 μmol/g, and 0.2–11.6 μmol/g, respectively. Additionally, stable isotope and elemental ratio analyses indicated that δ^13^C, δ^15^N, and C/N ratio values fell within the ranges of −29.6‰ to −22.4‰, 1.10‰ to 10.96‰, and 9.4‰ to 27.3‰, respectively. In general, temperature, silt proportion, sand proportion, and C/N ratio tended to increase with sediment depth. In contrast, salinity, moisture, TOC, TN, TP, IP, OP, δ^13^C, and δ^15^N declined along the vertical depth gradient. Differing from these monotonic trends, pH and clay content displayed fluctuating patterns as depth increased.

### 3.2 Vertical Variations in Archaeal Community Abundance and Taxonomic Composition

**FIGURE 2:**
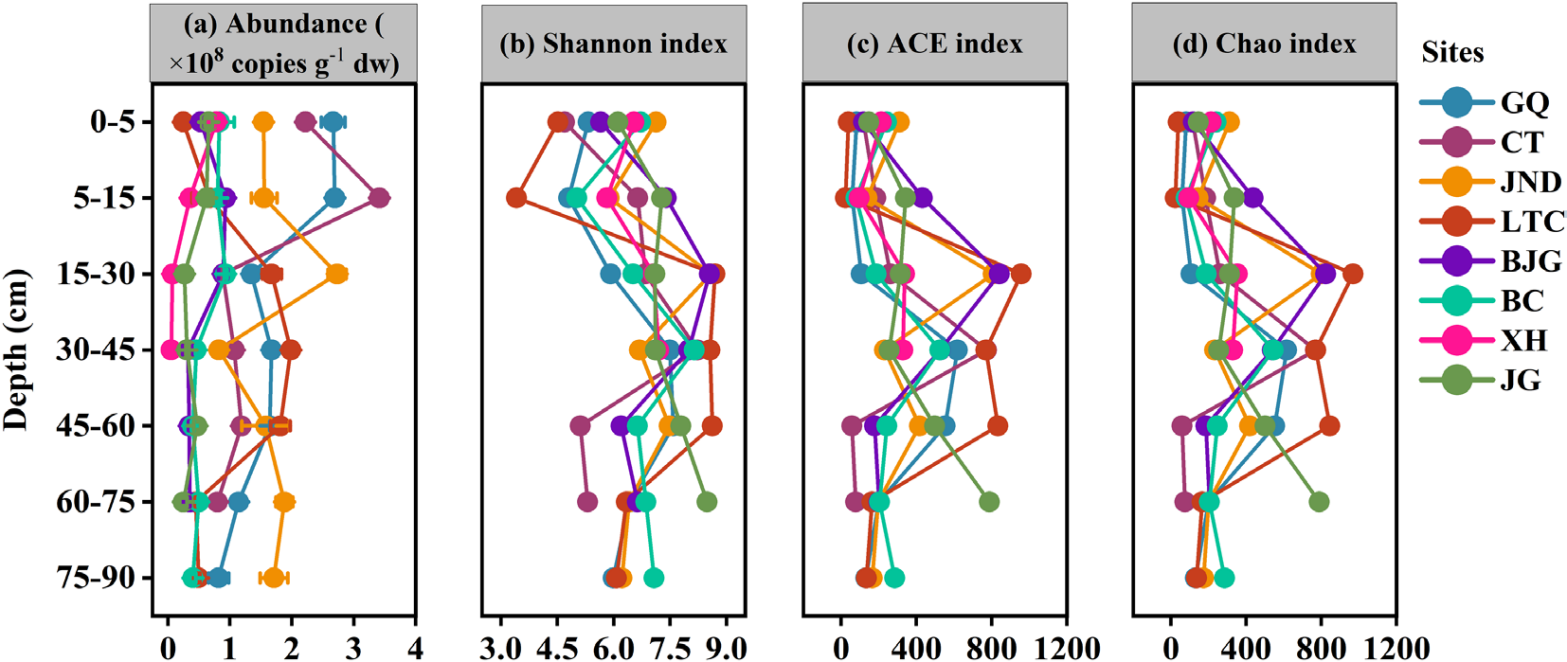
The vertical distribution of gene copy number and alpha diversity indices of archaeal communities in the surface and deep sediments of Zhanjiang mangrove wetlands. (a) Archaeal abundance, (b) Shannon index, (c) ACE index, and (d) Chao index.

The archaeal absolute abundance across all sediment samples ranged from 5.19 × 10^6^ to 3.41 × 10^8^ copies g^−1^ dw, with the maximum value observed at CT–15 cm and the minimum at XH–45 cm. Overall, archaeal abundance displayed a decreasing trend along the sediment depth gradient (Figure 2).

After quality filtering, a total of 5,235,841 high‐quality archaeal reads were retrieved from the 50 samples, with an average of 104,717 reads per sample; these sequences were assigned to 23,852 archaeal amplicon sequence variants (ASVs).

Values of the Shannon, ACE, and Chao indices across all samples ranged from 3.4 to 8.7, 23.0 to 957.0, and 23 to 969.5, respectively. Both archaeal diversity (Shannon index) and richness (ACE and Chao indices) exhibited an increasing trend along the sediment depth gradient (Figure 2).

**FIGURE 3:**
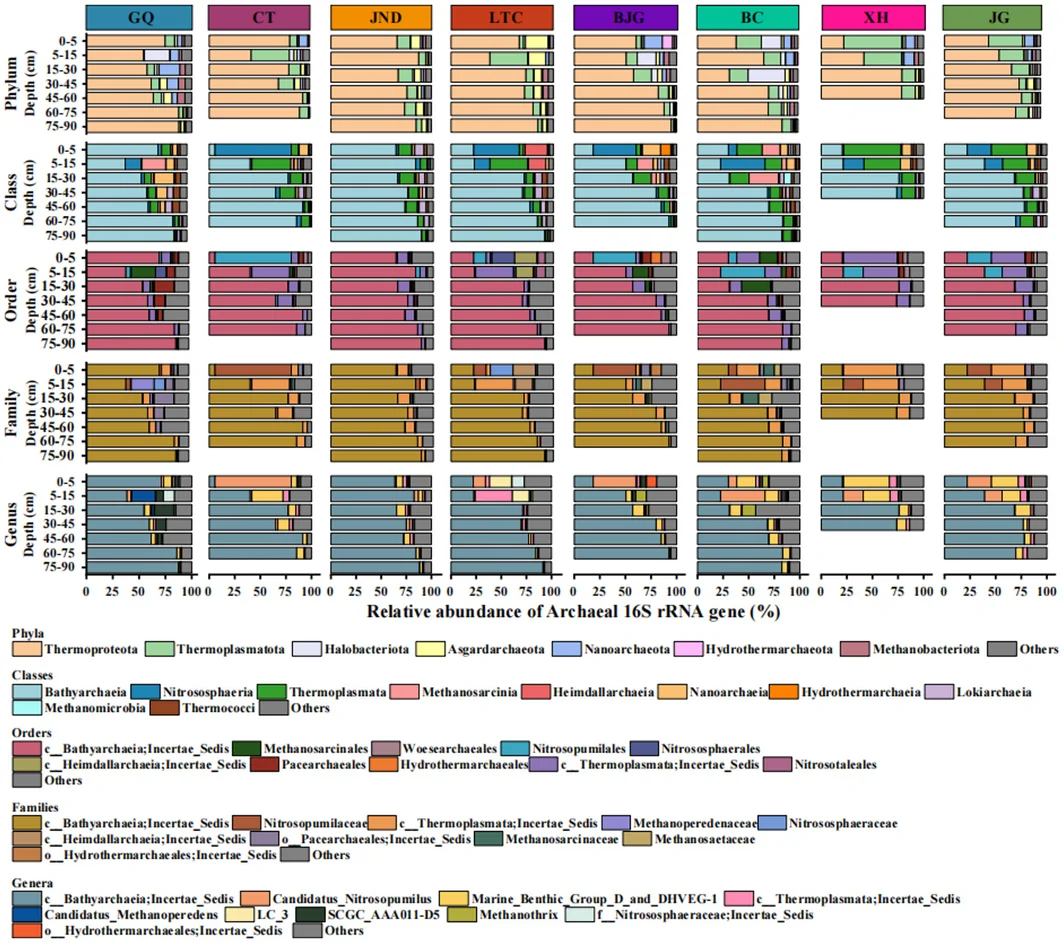
The taxonomic composition of archaeal community in the core sediments of Zhanjiang mangrove wetlands, at the phylum, class, order, family, and genus level, respectively.

**FIGURE 4:**
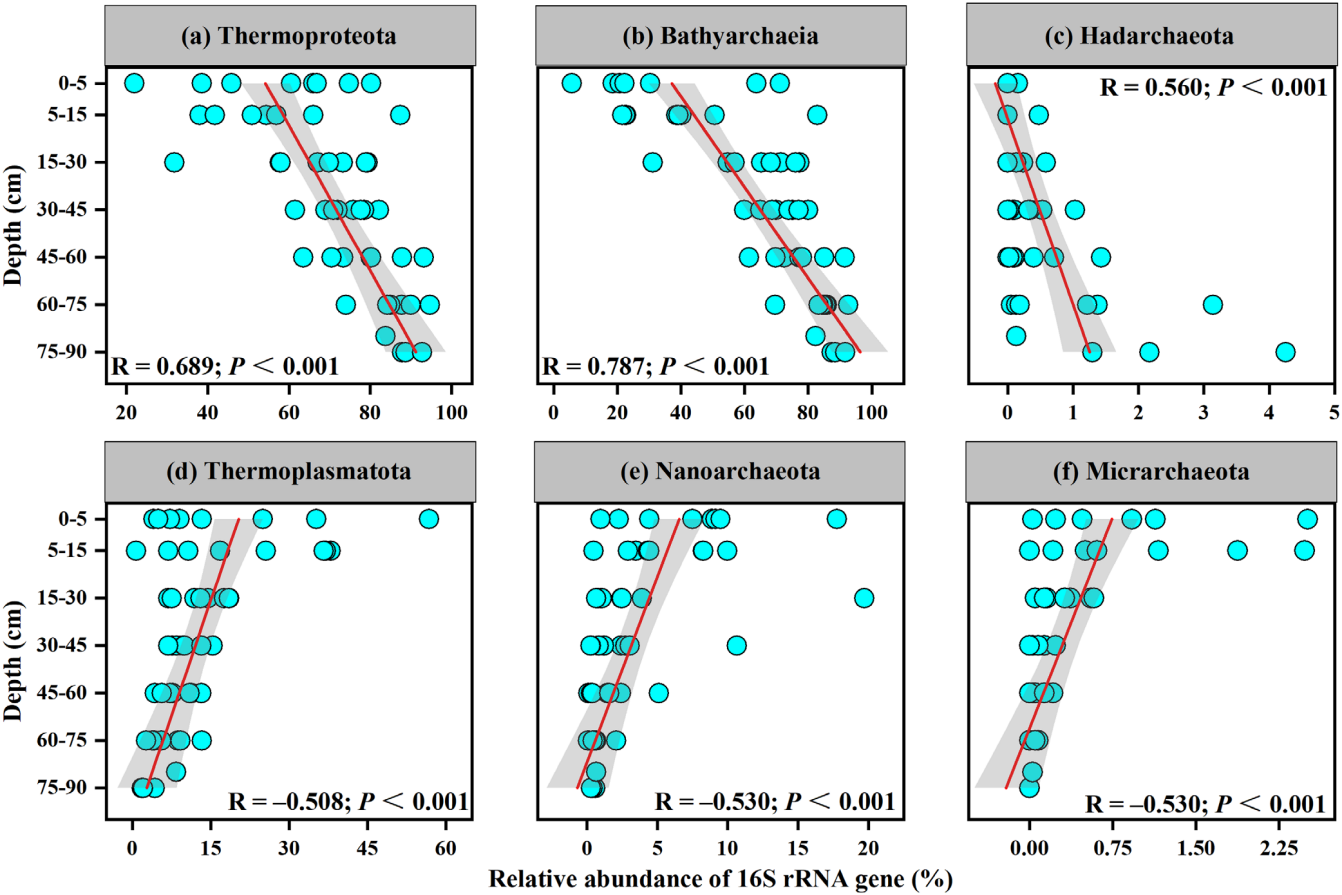
Changes in the relative abundance of dominant archaeal phyla (a and c–f) and class (b) across vertical mangrove sediment profiles. Linear regression models (red lines) quantified depth‐driven effects on these taxa, with gray shading indicating the 95% confidence interval (CI). The associated correlation coefficients (*R*) and *p* values are presented in each panel. Significant level: *p < 0.05; **p < 0.01; ***p < 0.001.

To characterize the vertical distribution of archaeal communities within mangrove sediments, community composition analyses were performed across five taxonomic ranks, including phylum, class, order, family, and genus (Figure 3). The archaeal community in surface sediments (0–15 cm) was mainly composed of three phyla: Thermoproteota (22.0%–87.4%), Thermoplasmatota (0.7%–56.8%), and Halobacterota (0.2%–24.1%), while Thermoproteota (31.8%–82.2%) and Halobacterota (0%–36.8%) emerged as the most abundant archaeal phyla in middle sediments (15–45 cm). In contrast, Thermoproteota (70.4%–94.7%) prevailed among archaeal taxa in deep sediments (45–90 cm), with Thermoplasmatota (1.8%–13.3%) as the subdominant group (Figure 3). Linear regression analysis revealed that the relative abundances of Thermoproteota and Hadarchaeota exhibited a significant increasing trend with sediment depth (*p* < 0.0001), whereas those of Thermoplasmatota, Nanoarchaeota, and Hydrothermarchaeota decreased markedly with depth (*p* < 0.05) (Figure 4).

At the class level, surface sediment archaeal assemblages featured abundant Bathyarchaeia (5.7%–82.8%), Nitrososphaeria (0.2%–74.5%), and Thermoplasmata (0.7%–56.0%), whereas Bathyarchaeia (31.2%–80.1%) and Methanosarcinia (0%–28.6%) attained the highest abundances among archaeal classes in middle sediments. In deep sediment layers, Bathyarchaeia (61.5%–92.6%) and Thermoplasmata (1.8%–13.3%) formed the major component of archaeal communities (Figure 3). Linear regression indicated that the relative abundance of Bathyarchaeia increased as sediment depth increased (*p* < 0.0001) (Figure 4). At the genus level, *Incertae Sedis* (5.7%–82.8%), *Candidatus Nitrosopumilus* (0%–74.5%), and *Marine Benthic Group D/DHVEG‐1* (*MBG‐D/DHVEG‐1*) (0%–44.8%) prevailed in surface sediment archaeal communities, while *Incertae Sedis* (31.2%–80.1%) and *SCGC AAA011‐D5* (0.1%–18.8%) occupied the most abundant genera in middle sediments. By comparison, *Incertae Sedis* (61.5%–92.6%) and *MBG‐D/DHVEG‐1* (0.3%–9.6%) comprised the core components of archaeal communities in deep sediments (Figure 3).

**FIGURE 5:**
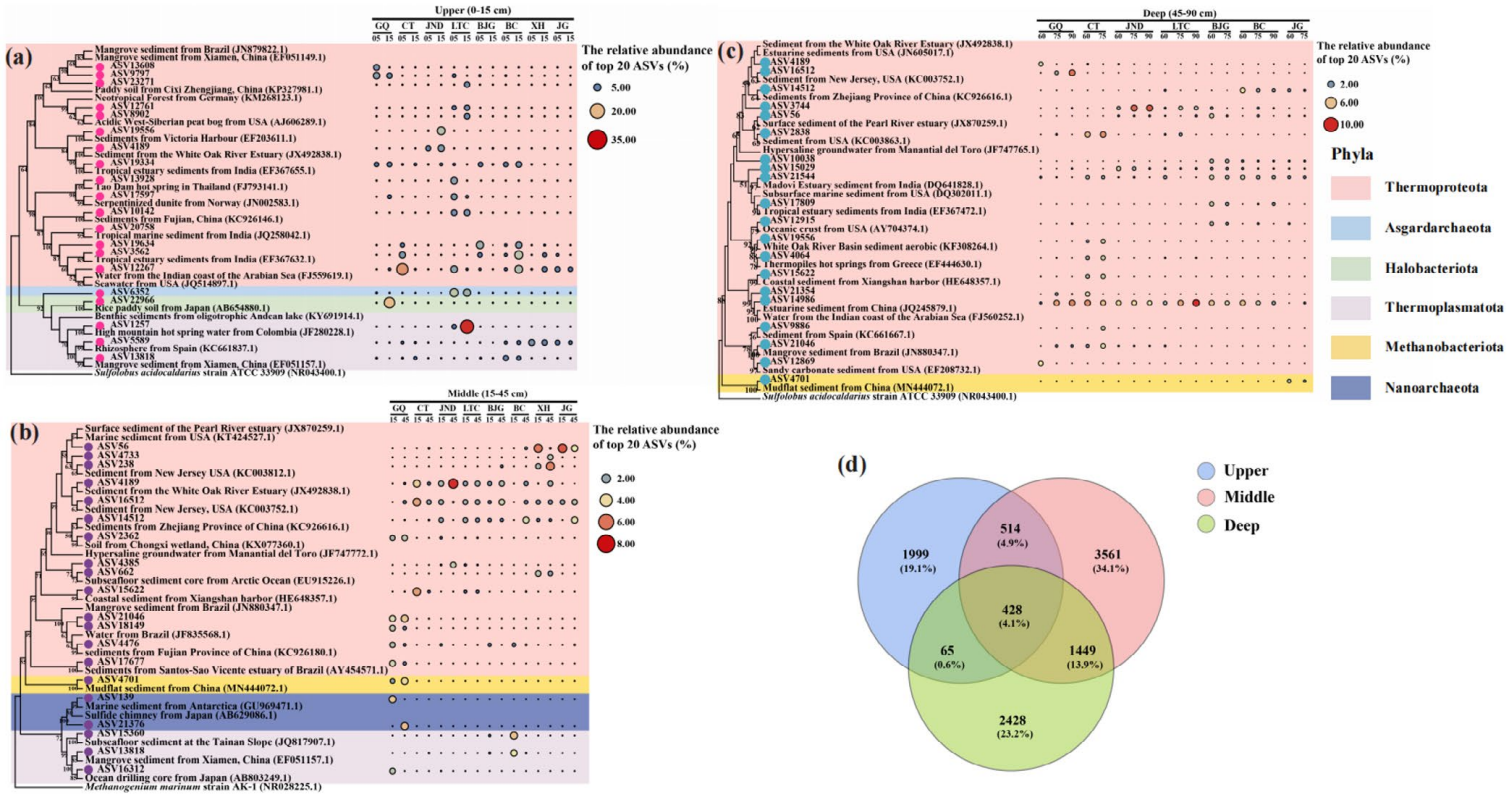
(a‐c) Phylogenetic trees of the top 20 most abundant archaeal ASVs in surface, middle, and deep sediments across the Zhanjiang mangrove wetlands, with their corresponding relative abundances presented in the bubble chart on the right. Four gradient scales of the bubble chart are shown on the left; Red and blue bubbles indicate ASVs with high and low relative abundances, respectively. ASVs affiliated with six distinct archaeal phyla are highlighted in different colors. (d) A Venn diagram showing the unique and shared ASVs among surface, middle, and deep sediments of the Zhanjiang mangrove wetlands.

To uncover the phylogenetic relationships of archaeal assemblages across three sediment depth layers in mangrove wetlands, the top 20 most abundant ASVs separately retrieved from surface, middle, and deep sediment layers were selected to construct three independent phylogenetic trees (Figure 5a–c). Phylogenetic analysis revealed that these ASVs from all three layers were affiliated with six archaeal phyla: Thermoproteota, Asgardarchaeota, Halobacterota, Thermoplasmatota, Methanobacteriota, and Nanoarchaeota (Figure 5a–c and Table S1).

Most recovered ASVs belonged taxonomically to Thermoproteota. However, several rare archaeal taxa exhibited strict layer‐specific distribution. For instance, ASV 6352 (Asgardarchaeota) and ASV 22966 (Halobacterota) were exclusively detected in surface sediments. ASV 6352 reached a maximum relative abundance of 12.2% at the LTC sites (0–5 cm) (Table S1). ASV 22966 exhibited a peak relative abundance of 19.6% at the GQ sites (5–15 cm), and shared 100.0% sequence similarity with an uncultured archaeon clone from Japanese rice paddy soil (Table S1).

Members of Nanoarchaeota were only identified in middle sediments. Specifically, ASV 139 peaked at a relative abundance of 4.4% at the GQ sites (15–30 cm), displaying 94.9% sequence similarity with an uncultured euryarchaeote clone from Antarctic marine sediments. Another nanoarchaeotal ASV (ASV 21376), which attained a maximum abundance of 4.5% in the GQ–45 cm layer, showed 93.0% sequence similarity to an uncultured archaeon clone from Japanese sulfide chimneys.

In contrast, ASV 1257 and ASV 15360 (affiliated with Thermoplasmatota) inhabited both surface and middle sediment layers; for example, ASV 1257 reached a peak relative abundance of 31.8% at the LTC site (5–15 cm) and shared 99.5% sequence similarity with the uncultured archaeon clone from a hot spring in Colombia. ASV 15360 attained a maximum relative abundance of 4.4% at the BC site (15–30 cm) and exhibited 98.4% sequence similarity to an uncultured archaeon clone from subseafloor sediments of the Tainan Slope.

Additionally, the phylum Methanobacteriota (represented by ASV 4701) was restricted to middle and deep sediment layers. This ASV achieved its highest relative abundance (4.0%) at the GQ site (30–45 cm) and exhibited 98.4% sequence similarity to the uncultured archaeon clone from Chinese mudflat sediments.

Venn diagram analysis revealed that 3006, 5952, and 4370 archaeal ASVs were recovered from the surface, middle, and deep sediment layers, respectively. The surface, middle, and deep layers harbored 1999, 3561, and 2428 unique archaeal ASVs, respectively. Only 428 ASVs (4.1% of the total combined ASVs across three depth groups) were shared across all three layers, suggesting limited overlap of archaeal ASVs across vertical sediment profiles (Figure 5d).

### 3.3 Vertical Compositional Dissimilarities Among Archaeal Communities Across Different Sediment Layers

**FIGURE 6:**
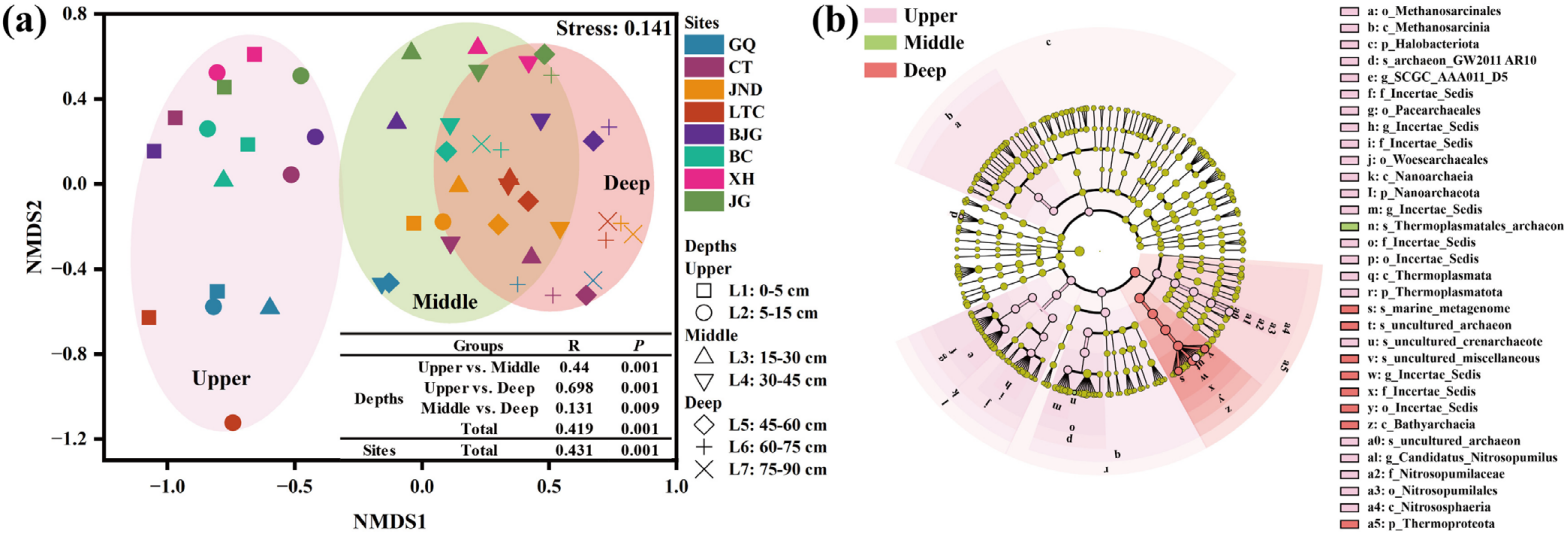
(a) Non‐metric multidimensional scaling (NMDS) analysis and analysis of similarities (ANOSIM) of archaeal communities based on Bray‐Curtis distance metrics, partitioned by geographical location and sediment depth in Zhanjiang mangrove wetlands. (b) Linear discriminant analysis effect size (LEfSe) analysis of archaeal communities in surface, middle, and deep sediments of Zhanjiang mangrove wetlands.

Non‐metric multidimensional scaling (NMDS) and analysis of similarities (ANOSIM) uncovered significant divergence in archaeal community composition among distinct sampling sites (*R* = 0.431; *p* = 0.001) and sediment layers (*R* = 0.419; *p* = 0.001) (Figure 6a).

LEfSe analysis detected a total of 23, 1, and 8 differentially abundant archaeal biomarkers in surface, middle, and deep sediments, respectively (Figure 6b). Archaeal indicator taxa exhibited the highest diversity in surface sediments, mainly comprising members of Thermoproteota, Iainarchaeota, Nanoarchaeota, Thermoplasmatota, and Halobacterota. In contrast, *Thermoplasmatales archaeon* was the sole archaeal biomarker recovered from middle layers. Furthermore, Thermoproteota, Incertae Sedis, Bathyarchaeia, *uncultured miscellaneous lineages*, *uncultured archaeon*, and *marine metagenomic groups* constituted the archaeal indicator assemblage in deep sediments (Figure 6b).

### 3.4 Vertical Variations in Metabolic Functional Predictions of Archaeal Communities

**FIGURE 7:**
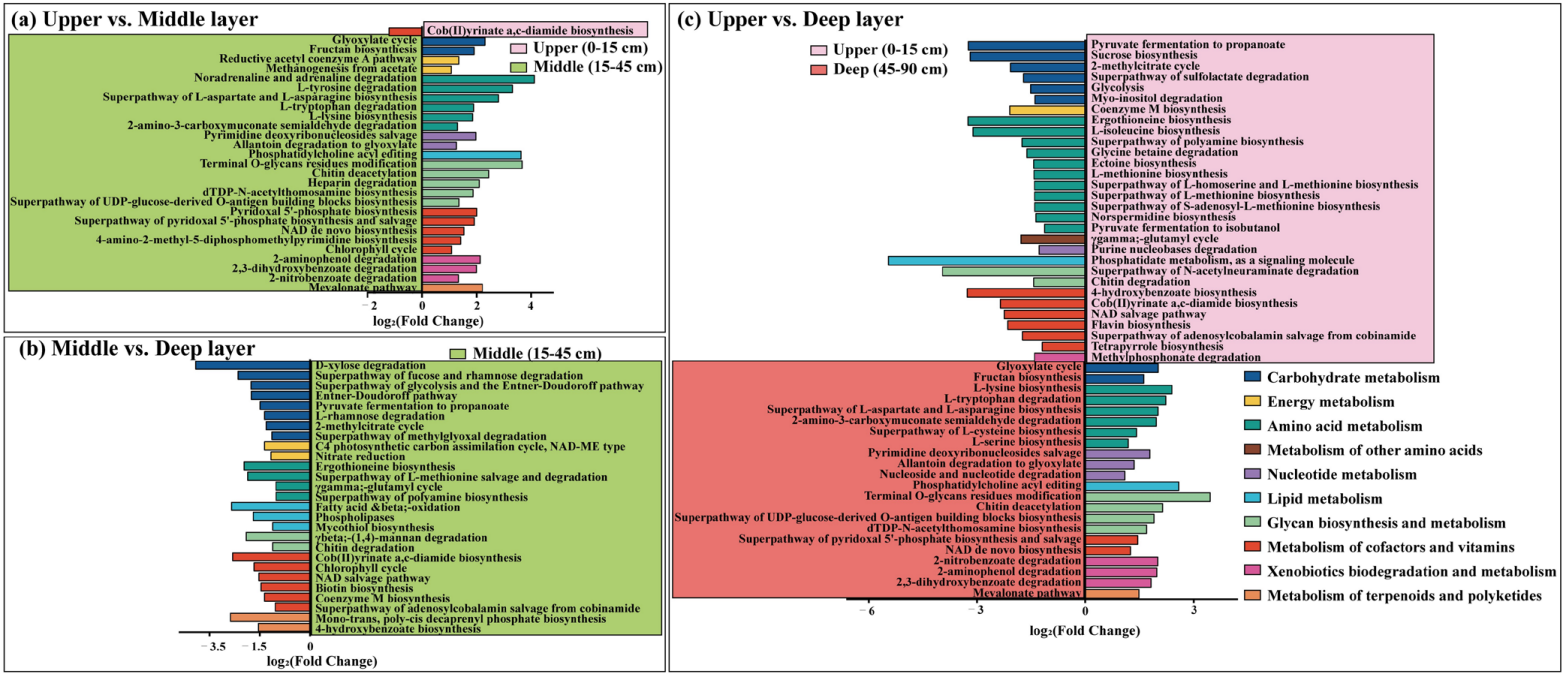
Vertical variations in functional predictions of archaeal communities along sediment depth gradients in Zhanjiang mangrove wetlands.

PICRUSt2‐based functional predictions revealed that archaeal communities harbored numerous differentially abundant metabolic genes across surface, middle, and deep sediment layers (Figure 7). Compared with those in surface and deep layers, archaeal communities in middle sediments displayed versatile metabolic capacity, with 54 unique metabolic pathways detected, covering carbohydrate metabolism, amino acid metabolism, metabolism of cofactors and vitamins, and glycan biosynthesis and metabolism (Figure 7a,b).

Relative to deep sediments, surface sediment samples contained 30 discrete metabolic pathways dominated by carbohydrate metabolism, amino acid metabolism, and metabolism of cofactors and vitamins. In contrast, deep‐sediment archaeal communities possessed 22 distinctive metabolic pathways when contrasted with surface layers, mainly involving glycan biosynthesis and metabolism, xenobiotics biodegradation and metabolism, and nucleotide metabolism (Figure 7c).

### 3.5 Vertical Divergence in Archaeal Co‐Occurrence Networks

**FIGURE 8:**
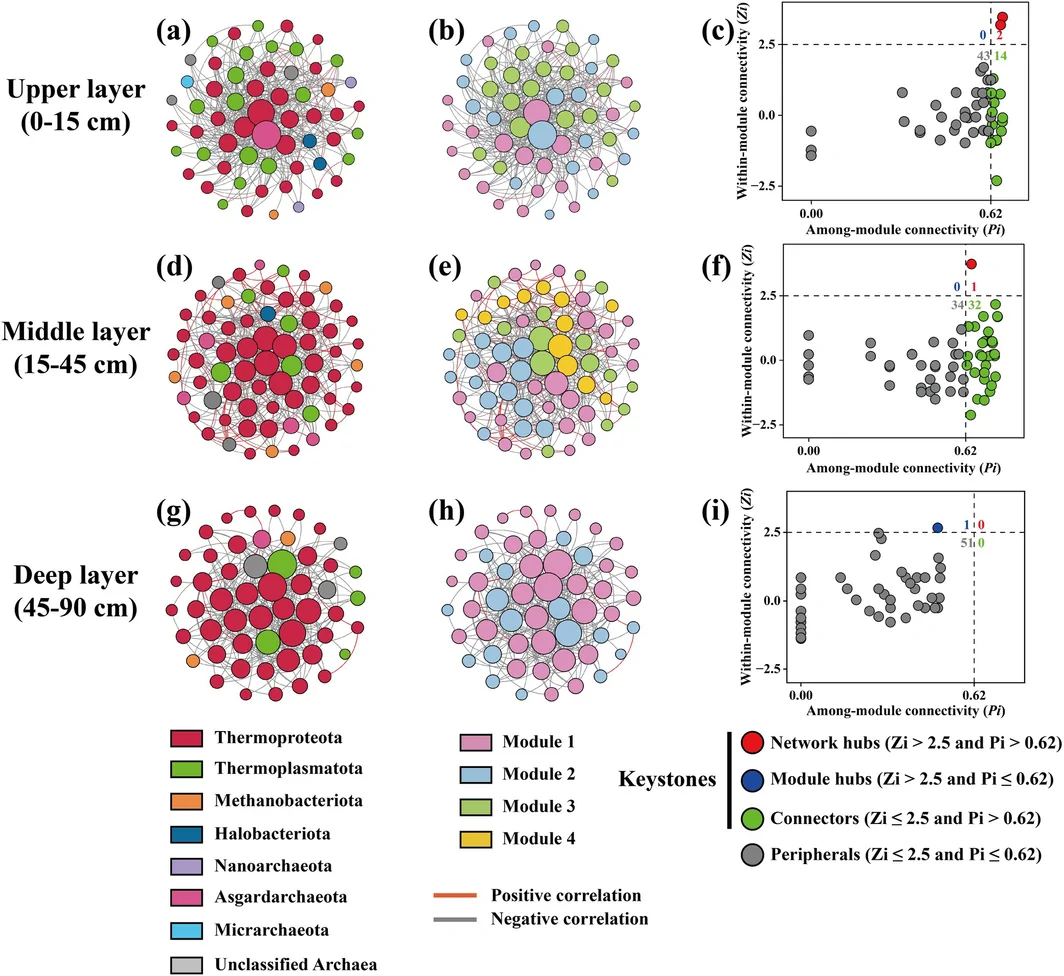
Co‐occurrence networks of archaeal communities in the surface, middle, and deep sediments of Zhanjiang mangrove wetlands. (a, d, g) Nodes are colored according to archaeal phyla. (b, e, h) Nodes are colored by different modules. (c, f, i) *Zi*‐*Pi* plots identifying putative keystone species.

To explore the variations in the inter‐taxa interactions of archaeal communities across the upper (0–15 cm, 16 samples), middle (15–45 cm, 16 samples), and the deep layers (45–90 cm, 18 samples), three co‐occurrence networks were established based on ASV profiles. The numbers of nodes and edges were 59 and 326, 67 and 342, and 52 and 233 for upper, middle, and deep networks, respectively. Notably, the percentage of negative links in the middle network (85.1%) was lower than those in the deep (97.4%) and surface (97.2%) networks, indicating higher stability of archaeal communities observed in the middle network. Furthermore, the surface network displayed greater complexity compared with the middle and deep networks, as evidenced by its elevated average connectivity (avgK), increased average clustering coefficient (avgCC), and reduced average path distance (GD). However, among the three networks, the middle network had the largest number of modules and the highest modularity values (Figure 8 and Table S2).

A total of 16 keystone taxa were identified in the surface network, including two network hubs and 14 connectors. The network hubs were affiliated with Thermoproteota and Asgardarchaeota, while the connectors belonged to Thermoproteota, Thermoplasmatota, and Nanoarchaeota. By comparison, 33 keystone taxa were detected in the middle network, consisting of one network hub and 32 connectors. This network hub was classified as Thermoproteota, whereas the connectors were assigned to Thermoproteota, Thermoplasmatota, Asgardarchaeota, Methanobacteriota, and Halobacteriota. Furthermore, the deep network harbored merely one module hub, taxonomically assigned to Thermoproteota (Figure 8 and Table S3).

### 3.6 Vertical Stratification of Archaeal Community Assembly Mechanisms

**FIGURE 9:**
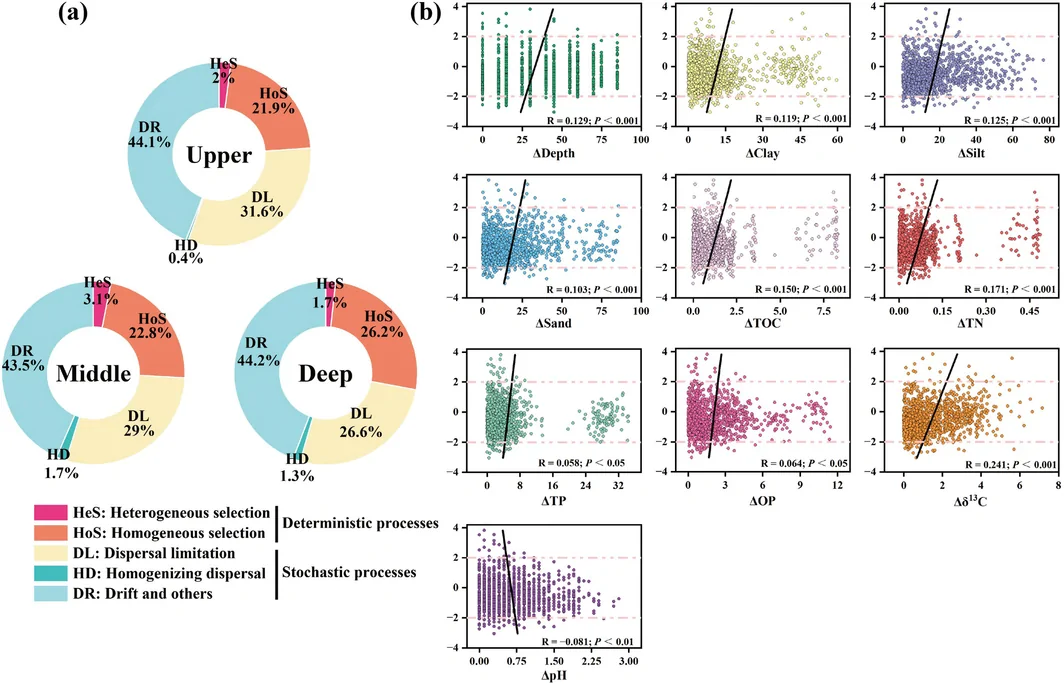
(a) Archaeal community assembly in the upper, middle, and deep sediments of Zhanjiang mangroves. (b) Relationships between β‐nearest taxon index (βNTI) values and ten physicochemical parameters (including sediment depths, sand content, silt content, clay content, TP, OP, TOC, δ^13^C, TN, and pH). Linear regressions models (black lines), correlation coefficients (R), and *p* values are displayed in each panel. Horizontal yellow dashed lines (βNTI values of 2 and −2) denote the significance thresholds.

The iCAMP results indicated that stochastic processes (particularly drift and dispersal limitation) predominated archaeal community assembly across surface (76.1%), middle (74.1%), and deep (72.1%) sediments. Likewise, the βNTI values of nearly all samples (accounting for 95.1% of all pairwise comparisons) ranged from −2 to 2, further demonstrating that stochastic processes governed archaeal community assembly throughout the vertical sediment column in mangroves. Meanwhile, βNTI values displayed a significant upward trend along the ΔDepth gradient (ΔDepth represents the absolute depth difference between any two sampling sites) (*R* = 0.129; *p* < 0.001), indicating that the proportion of deterministic processes increased with sediment depth. Furthermore, pH, clay, silt, sand, TOC, TN, TP, OP, and δ^13^C significantly regulated the archaeal assembly mechanisms in mangrove sediments (*p* < 0.05; Figure 9).

### 3.7 Key Environmental Drivers Governing the Vertical Distribution of Archaeal Communities

**FIGURE 10:**
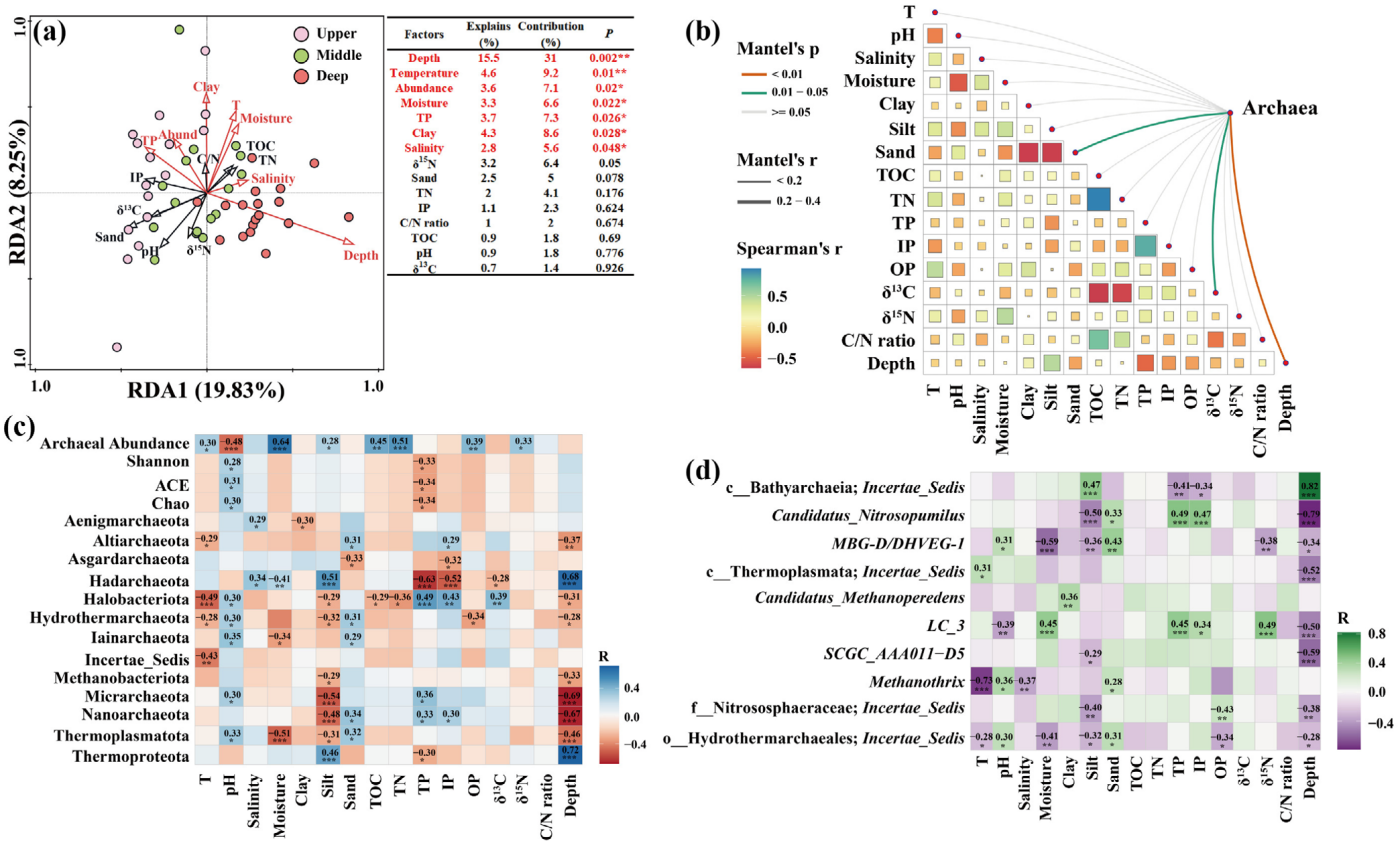
(a) Redundancy analysis (RDA) showing the relationships between archaeal community composition and environmental parameters in Zhanjiang mangrove wetlands. (b) Mantel test heatmap illustrating correlations between archaeal communities and sediment physicochemical parameters in Zhanjiang mangrove wetlands. Orange and green lines denote significant correlations (*p* < 0.01 and *p* < 0.05, respectively), while gray lines indicate no correlation. Line thickness corresponds to Spearman's correlation coefficients: Thicker lines represent stronger correlations, and thinner lines represent weaker correlations. (c–d) Correlation heatmap illustrating the relationship between dominant archaeal groups and sediment physicochemical properties in mangrove sediments.

RDA results showed that the two RDA axes together explained 28.08% of the total variation, with axis 1 accounting for 19.83% and axis 2 for 8.25%. These parameters explained 50.10% of the observed variability in the archaeal community. RDA analysis revealed that seven key environmental parameters, i.e., sediment depth (Explains = 15.5%, Contribution = 31.0%, *p* = 0.002), temperature (Explains = 4.6%, Contribution = 9.2%, *p* = 0.01), abundance (Explains = 3.6%, Contribution = 7.1%, *p* = 0.02), moisture (Explains = 3.3%, Contribution = 6.6%, *p* = 0.022), TP (Explains = 3.7%, Contribution = 7.3%, *p* = 0.026), clay (Explains = 4.3%, Contribution = 8.6%, *p* = 0.028), and salinity (Explains = 2.8%, Contribution = 5.6%, *p* = 0.048), were observed to significantly affect the mangrove archaeal communities (Figure 10a). Likewise, combined Mantel test and Spearman correlation analysis further demonstrated that sediment depth acted as the dominant environmental factor shaping archaeal community assembly in mangrove sediments (*p* < 0.01; Figure 10b).

Two Spearman's rank correlation heatmaps were generated to illustrate the relationships between all the archaeal phyla (or the top 10 genera) and sediment physicochemical parameters. The results demonstrated that Thermoproteota was significantly positively influenced by depth and silt, but negatively correlated with TP. Thermoplasmatota exhibited a statistically significant positive association with sand and pH, while showing a negative association with depth, silt, and moisture. In contrast, pH, TP, IP, and δ^13^C had a positive correlation with Halobacterota, yet were negatively correlated with depth, TN, TOC, silt, and temperature. Meanwhile, archaeal abundance was significantly positively correlated with temperature, moisture content, silt, TOC, TN, OP, and δ^15^N, while exhibiting a negative association with pH. The Shannon, ACE, and Chao indices showed pronounced positive relationships with pH, while possessing a negative correlation with TP (Figure 10c).

Additionally, the genus *Incertae Sedis* was positively associated with depth and silt, while negatively correlated with TP and IP. Another genus *Candidatus Nitrosopumilus* displayed a negative association with depth and silt, yet a positive correlation with sand, TP, and IP. Furthermore, *MBGD/DHVEG‐1* showed positive correlations with pH and sand, whereas negative associations were observed between these taxa and moisture, silt, depth, and δ^15^N (Figure 10d).

## 4 Discussion

### 4.1 Depth‐Dependent Shifts in Composition and Metabolic Potential of Archaeal Communities in Mangrove Sediments

In the present study, the archaeal abundance declined progressively as sediment depth increased (Figure 2a), which may be attributed to a reduction in terms of sediment porosity and gradual mineralization of soil organic matter along the depth gradient (Zhong et al. 2025). Similar findings were observed in mangrove sediments from Tieshan Bay, China, where archaeal abundance showed a gradual reduction across the 0–20 cm sediment profile (Wang, Zheng, Xing, et al. 2024). In contrast, total archaeal abundance displayed an increasing trend with sediment depth in mangrove wetlands from Futian, China (0–40 cm) and Qi'ao Island (0–100 cm) (Ding et al. 2025; Qi et al. 2025). Conversely, the archaeal abundance in this study was significantly affected by T, pH, moisture content, silt, TOC, TN, OP, and δ^15^N (Figure 10c), aligning with previous studies reported that archaeal abundance in mangrove sediments is influenced by sediment depth, particle size, temperature, C/N ratio, organic matter content, and organic pollutants (Militon et al. 2024; Wang, Zheng, Xing, et al. 2024; Zhang, Chen, Sun, et al. 2021; Zhang, Pan, et al. 2021).

In contrast, archaeal diversity exhibited an overall rising trend as sediment depth increased in this study (Figure 2b–d), a pattern consistent with observations from prior surveys in Singapore mangroves (100 cm) (Ng et al. 2025); seven typical mangrove nature reserves in Southeast China (0–30 cm) (Zhang, Pan, et al. 2021); and Hong Kong mangrove reserve (0–45 cm) (Zhou et al. 2017). However, the archaeal diversity decreased with depths in Tieshan Bay mangrove (0–20 cm) (Wang, Zheng, Xing, et al. 2024). Two primary factors accounted for the elevated archaeal diversity in deeper sediments: (1) vertical stratification of microhabitats, driven by depth gradients of oxygen penetration, redox potential, and organic matter lability within sediments, results in differentiation of archaeal diversity across sediment depths (Besseling et al. 2018); (2) the inherent tolerance of archaea to low energy availability and their preferential utilization of degraded buried organic matter (Gong et al. 2022; Hoehler and Jørgensen 2013). Additionally, our analyses demonstrated that the archaeal diversity was positively correlated with pH, whereas a negative association was detected between this diversity metric and TP (Figure 10c). Moreover, gravel proportion, N/NO_3_^−^, total sulfur, and N/NH_4_^+^ have been documented to impose a marked effect on archaeal diversity of mangrove sediments (Zhang, Pan, et al. 2021). Nevertheless, we should acknowledge one analytical limitation relevant to our alpha‐diversity estimates. We acknowledge that our rarefaction depth (5110 sequences per sample) is relatively low for high‐throughput microbiome investigations. While this sequencing depth reliably recovered dominant archaeal taxa and core community assembly patterns across the three sediment layers, it may restrict the detection of rare, low‐abundance taxa and subtle differences in alpha diversity.

Our results revealed distinct vertical stratification of archaeal assemblages across mangrove sediment depth gradients (Figures 3, 4 and 6). Thermoproteota prevailed in the surface sediment layer, while Thermoproteota and Halobacteriota constituted the dominant archaeal taxa in the middle sediment layer. Thermoproteota (notably the subgroup Bathyarchaeia) exclusively occupied deep sediment layers (Figure 3).

Similar findings have been reported in previous studies (Luis et al. 2019; Meng et al. 2022; Pan et al. 2019; Wang, Zheng, Xing, et al. 2024; Zhou et al. 2017). In detail, Pan et al. reported that Bathyarchaeota exhibited greater relative prevalence in the deep sediments (38 cm) of Futian mangrove wetland (Pan et al. 2019). Wang et al. verified that Bathyarchaeia and Thermoplasmatota exhibited markedly elevated relative abundance within the 0–20 cm mangrove sediment profile of Tieshan Bay, China (Wang, Zheng, Xing, et al. 2024). Moreover, in seven South China mangroves, Crenarchaeota and Euryarchaeota were more abundant in surface layers (0–5 cm), while Asgardarchaeota dominated subsurface sediments (25–30 cm) (Meng et al. 2022). Thaumarchaeota Marine Group I constituted the major archaeal group in surface sediments (0–2 cm) while Bathyarchaeota and MBG‐B dominated in subsurface sediments (10–45 cm) in Mai Po Nature Reserve (Zhou et al. 2017). Likewise, Euryarchaeota and Lokiarchaeota occupied the largest proportion within surface sediments (0–10 cm), whereas Bathyarchaeota and Lokiarchaeota prevailed in deep layers (40–50 cm) of Saint Vincent Bay mangroves (Luis et al. 2019).

The dominance of Bathyarchaeia in deep sediments observed in this study can be explained by its strong environmental adaptability. In agreement with this observation, previous investigations have repeatedly confirmed that Bathyarchaeia is among the most ubiquitous and prevalent archaeal groups across natural habitats, with an estimated global cell abundance of 2.0–3.9 × 10^28^ (Pan et al. 2019). This archaeal group thrives across diverse anoxic subsurface environments, spanning marine (Dong et al. 2025; Ishaq et al. 2024), mangrove (Ni et al. 2024; Wang, Zheng, Ni, et al. 2024; Wang, Zheng, Xing, et al. 2024), estuarine (Romano et al. 2021; Yue et al. 2022), freshwater (Ruiz‐Blas et al. 2024; Zhao et al. 2025), soil ecosystems (Bandla et al. 2024), hot springs (Qi et al. 2021), hydrothermal vents (Su et al. 2023), and cold seeps (Ruff et al. 2015).

In the present study, archaeal communities exhibited distinct metabolic differences between surface and deep sediment layers. More specifically, surface archaeal communities primarily carried out carbohydrate metabolism, amino acid metabolism, and metabolism of cofactors and vitamins, whereas glycan biosynthesis and metabolism, xenobiotics biodegradation and metabolism, and nucleotide metabolism constitute the predominant metabolic pathways in deep archaeal communities (Figure 7). Substantial prior literature has documented distinct vertical stratification of metabolic potentials in microbial communities (Bhattacharya et al. 2025; Gao et al. 2025; Ren et al. 2025; Wang, Zheng, Xing, et al. 2024). Wang, Zheng, Xing, et al. (2024) reported that archaeal communities exhibited enhanced functional potential for carbohydrate metabolism, amino acid metabolism, and metabolism of cofactors and vitamins in surface sediments (0–15 cm) of Tieshan Bay mangroves. Ren et al. (2025) demonstrated that the overall potential of methane metabolism within archaeal communities decreased with depth in sediments from the Qinghai‐Tibet Plateau. Meanwhile, Gao revealed a depth‐dependent reduction in nitrogen metabolism potential for sediment microbial communities in Honghu Lake (Gao et al. 2025). Bhattacharya et al. (2025) documented that xenobiotics biodegradation and metabolism occurred in archaeal assemblages from surface sediments of the Indian Sundarban mangrove ecosystem. Collectively, these observations could be explained by two interrelated ecological processes: (i) pronounced shifts in oxygen concentration, redox potential, and organic matter composition along the sediment depth gradient (Huang et al. 2026; Qi et al. 2025; Rodríguez‐Ramos et al. 2021; Yuan et al. 2026); (ii) the replacement of surface‐adapted archaea by anaerobic specialists and subsequent shifts in dominant metabolic processes (Ding et al. 2025; Du et al. 2022; Sheikh et al. 2025).

Further correlation analysis demonstrated that the relative abundance of Thermoproteota exhibited strong positive associations with sediment depth and silt content, whereas it was markedly negatively correlated with TP (Figure 10c). Numerous environmental factors have been documented to regulate the distribution of Bathyarchaeia distribution (Fillol et al. 2016), including salinity (Zou et al. 2020), temperature (Berben et al. 2022; Qi et al. 2021; Su et al. 2023), oxygen availability (Hoshino et al. 2020; Yi et al. 2024), TOC (Li et al. 2020; Loh et al. 2021; Nesbø et al. 2024; Pelsma et al. 2022; Zhang, Pan, et al. 2021), TN (Meng et al. 2022), season (Zhang et al. 2025), and heavy metal concentrations (Bhattacharya et al. 2025). On the other hand, Thermoplasmatota displayed positive associations with pH and sand content, while showing significant negative relationships with depth, silt, and moisture content (Figure 10c). This pattern aligns with prior research: most taxa within Thermoplasmatota are facultative anaerobes capable of thriving in microoxic sediment habitats via fermentative or mixotrophic metabolism (Mu et al. 2025; Mubaraq et al. 2024; Zhou et al. 2018).

### 4.2 Depth Shapes Interaction Patterns of Archaeal Assemblages in Mangrove Sediments

Within natural ecological systems, microorganisms tend to assemble into complex interactive ecological networks (Banerjee et al. 2019; Guseva et al. 2022; Ma et al. 2020). Co‐occurrence network analysis enables the detection of intricate interspecific interactions and the ecological significance of keystone species (Brauner et al. 2025; Lai et al. 2025; Ren et al. 2021; van Vliet et al. 2022; Wang, Zheng, Xing, et al. 2024; Zhang, Pan, et al. 2021). After comparing co‐occurrence networks across surface, middle and deep sediments, we found that the surface network exhibited higher topological complexity, whereas the middle network possessed superior structural stability (Figure 8). These observations are consistent with previous findings reported for surface sediments (0–10 cm) of the Bohai Sea (Gong et al. 2022) and middle soil layers (20–50 cm) across China (Yokota et al. 2022). Alternatively, Qian et al. documented that the mid‐layer network (15–30 cm) showed the greatest complexity as opposed to the surface (0–15 cm) and deep (30–100 cm) sediment networks within Qi'ao Mangrove Reserve (Qian et al. 2023). The divergent vertical patterns of microbial co‐occurrence networks observed across mangrove sites (Zhanjiang and Qi'ao Island) are primarily attributable to disparities in dominant mangrove vegetation, sedimentary and tidal regimes, and vertical geochemical stratification (including redox conditions, sulfide levels and substrate properties) (Chen and Wen 2021; Qian et al. 2023; Zhang, Pan, et al. 2021).

The greater complexity of the surface network can be explained by two mechanisms: (1) surface sediments were enriched in labile organic matter that sustained highly diverse and abundant microbial communities (Shilla 2021), thereby contributing to the elevated network complexity observed in surface layers (Fu et al. 2020; Gong et al. 2022; Pan et al. 2019); (2) meanwhile, the oligotrophic and anaerobic extreme environment of deep sediments exerted intense environmental stress, which subsequently diminished microbial interactions (Jørgensen and Marshall 2016; Zhang, Yao, Sun, et al. 2021).

These observations of greater stability in middle sediments networks may be ascribed to the following interconnected factors: (1) The middle sediment layer (15–45 cm) lies precisely in the transition zone between the surface layer with intense oxidative disturbance and the restrictive anoxic deep layer, providing habitable niches for archaea with divergent metabolic functions (Du et al. 2022; Zhao et al. 2020); (2) the ample energy supplied in middle sediments enhanced functional redundancy, which consequently reinforced the ecological stability of archaeal assemblages (Cornell et al. 2023; Lin et al. 2025).

Notably, the proportion of negative associations in the deep network was the highest among the three networks (Figure 8 and Table S2), indicating stronger antagonistic interactions in deeper sediment zones.

This phenomenon could be ascribed to two primary mechanisms: first of all, increasing sediment depth induced a sharp decline in bioavailable nutrients (e.g., oxygen, dissolved organic carbon, nitrogen, and phosphorus) and resource scarcity consequently drove competitive interactions among distinct archaeal taxa (Qian et al. 2023); secondly, methanogenic archaea tend to be highly enriched in deep sediment horizons, with the competition or predation between these groups gives rise to pronounced negative correlations (Qian et al. 2023). This phenomenon can be mainly attributed to the reduction in bioavailable nutrients along the sediment depth gradient and the enrichment of methanogenic archaea in deep horizons, which collectively intensify intertaxon competition or predation, thereby generating pronounced negative correlations (Qian et al. 2023).

Keystone taxa are microbial groups that are highly connected within the network and play pivotal roles in maintaining the structural organization and functions of archaeal assemblages (Chen and Wen 2021; Ma et al. 2020; Meng et al. 2023; Ng et al. 2025; Xun et al. 2021). Network hubs sustain microbial community stability and functional performance; module hubs construct network architectures and maintain network stability, whereas connectors mainly serve as mediators facilitating information processing and transfer within networks (Deng et al. 2012; Liu et al. 2022; Purahong et al. 2024; Qian et al. 2023). Our study revealed a shift from surface networks with multiple network hubs, through connector‐dominated middle‐layer networks, to deep networks containing only one module hub (Figure 8).

Thermoplasmatota, Nanoarchaeota, Methanobacteriota and Halobacteriota served as connectors between surface and middle layers, mainly facilitating cross‐module communication to maintain network stability. Asgardarchaeota shifted from network hubs in surface sediments, which preserved overall network stability and functional integrity, to connectors in middle layers responsible for cross‐module bridging. Thermoproteota acted as both network hubs and connectors in surface and middle layers, and constituted the sole module hub within deep sediment layers. This phylum guaranteed overall network stability, functional integrity and cross‐module communication in surface and middle sediments, while supporting local network organization and functional specialization in deep horizons. This pattern could largely be attributed to the diverse metabolic capabilities of archaeal communities, which triggered transitions in keystone taxa identity along the sediment depth gradient (Hager et al. 2025; Hassani et al. 2023; Hou et al. 2025; Qian et al. 2023; Slosser et al. 2025; Sun et al. 2025; Zhao et al. 2022; Zheng et al. 2022).

### 4.3 Stochastic Processes Dominate Mangrove Archaeal Community Assembly

Stochastic and deterministic processes were two of the most thoroughly characterized ecosystem processes governing the structuring patterns of microbial communities, which may dominate in diverse environments, or both may be equally important (Li et al. 2024; Zhang, Pan, et al. 2021). A wealth of investigations has suggested that homogeneous selection (Ni et al. 2024; Zhang, Pan, et al. 2021), heterogeneous selection (Zhang et al. 2019; Zhou et al. 2024), dispersal limitation (Liu et al. 2020, 2023; Ni et al. 2024), homogenizing dispersal (Chen and Wen 2021), and drift (Chen and Wen 2021) respectively dominated the structuring processes of the microbial community within various disparate mangrove habitats.

In this study, iCAMP and βNTI analyses revealed that stochastic processes governed archaeal community assembly in surface, middle and deep sediments of the Zhanjiang mangrove wetlands (Figure 9). Importantly, we did not detect significant vertical shifts in the relative importance of assembly processes among the three depth groups along the 0–90 cm sediment profile. The results of the present study align with conclusions drawn from multiple earlier studies, for instance, stochastic processes accounted for 93.8% of prokaryotic community assembly in seven representative mangrove nature reserves (Zhang, Pan, et al. 2021). In another study, stochastic processes outweighed deterministic processes on shaping microbial community structures in *Spartina alterniflora* ‐invaded subtropical mangrove sediments (Chen and Wen 2021). Additionally, stochastic processes function as the dominant drivers governing the assembly of methanogenic assemblages in pristine mangrove ecosystems (Li et al. 2024). Nevertheless, deterministic processes exert a pivotal regulatory role in assembly processes of archaeal communities in several typical mangroves from China (Zhang, Pan, et al. 2021). This discrepancy might be ascribed to variations in growth habits, metabolic diversity, dispersal capacities, and sampling scales (Gutiérrez‐Preciado et al. 2024; Rain‐Franco et al. 2024).

Notably, correlation analyses uncovered significant relationships between βNTI and multiple sediment geochemical variables, indicating strong environmental control on archaeal community variation despite the overall dominance of stochastic assembly (Sun et al. 2023; Zhang, Pan, et al. 2021). We propose a hierarchical framework to explain this pattern: persistent environmental filtering first defines the viable taxonomic pool by selecting taxa adapted to local sediment conditions (Wang, Zheng, Ni, et al. 2024; Zhang, Pan, et al. 2021), while fine‐scale microhabitat heterogeneity, dispersal limitation, and ecological drift determine the final community composition within this species pool (Chen and Wen 2021). In short, environmental constraints define the range of potential colonists, and stochastic dynamics shape fine‐scale community variation within the suitable niche space (Wu et al. 2022; Zhang, Pan, et al. 2021).

The links between βNTI values and variations in biogeochemical parameters were utilized to identify the dominant drivers of archaeal community assembly within mangrove habitats. Except depth, our results also indicated that sediment silt content, sand content, clay content, TP, OP, TOC, TN, and δ^13^C were statistically positively correlated with βNTI values, whereas pH presented a marked negative correlation with βNTI values (Figure 9). Recent research has also revealed that depth and nutrient regimes are core factors governing sediment prokaryotic community assembly in several typical mangroves from China (Zhang, Pan, et al. 2021) and in methane seepage regions (Zhong et al. 2025). Additionally, a previous study demonstrated that mean annual precipitation, TOC, salinity, nitrate, and TN exerted stronger influences on prokaryotic assembly processes in a range of mangrove habitats (Zhang et al. 2019). Li et al. (2024) further revealed that the βNTI values of methanogenic consortia were positively affected by nitrate concentrations in the Guangdong mangrove ecosystem.

## 5 Conclusion

The present research provides pioneering insights into how sediment depth shapes archaeal community structure, functional profiles, co‐occurrence network dynamics, along with community assembly processes within the Zhanjiang mangrove wetlands of Guangdong, China. These results revealed that archaeal abundance decreased with increasing sediment depth, whereas alpha diversity increased along the vertical depth gradient. Thermoplasmatota emerged as the dominant phylum in surface sediments, while Thermoproteota (specifically the class Bathyarchaeia) were substantially enriched in deep sediments. Functionally, surface archaeal communities are characterized by carbohydrate metabolism, amino acid metabolism, and metabolism of cofactors and vitamins, whereas glycan biosynthesis, xenobiotics biodegradation, and nucleotide metabolism constitute the predominant functional pathways in deep archaeal communities. The surface co‐occurrence network exhibited higher network complexity and structural stability compared to the deep counterpart, and Thermoproteota constituted the core keystone taxa in all three networks. Moreover, iCAMP and βNTI analyses suggested that stochastic processes strongly govern archaeal community assembly across the vertical sediment profile. Additionally, sediment depth, temperature, moisture, TP, clay content, and salinity were the major variables that significantly influenced archaeal community composition. The findings provide a theoretical and empirical basis for comprehensively elucidating the biogeochemical roles of archaeal communities in deep mangrove sediments.

## Author Contributions

**Yunru Li:** software (equal), visualization (lead), writing – original draft (lead). **Songze Chen:** methodology (equal), software (lead), writing – review and editing (equal). **Qing Li:** investigation (equal), visualization (equal). **Yue Sun:** visualization (equal). **Dan Sun:** writing – review and editing (equal). **Mingzhong Liang:** project administration (equal), supervision (equal). **Wei Zhang:** formal analysis (equal). **Jianqing Chen:** writing – review and editing (equal). **Jing Sun:** formal analysis (equal). **Bin Gong:** supervision (equal), writing – review and editing (equal). **Jingjing Song:** writing – review and editing (equal). **Ying Liu:** conceptualization (lead), data curation (equal), investigation (equal), methodology (equal), software (equal), supervision (lead), validation (equal), writing – review and editing (lead). **Rongping Bu:** project administration (lead), writing – review and editing (equal).

## Ethics Statement

The authors have nothing to report.

## Conflicts of Interest

The authors declare no conflicts of interest.

## Supporting information


**Table S1:** The taxanomy classification, NCBI reference sequences, isolation source, percent identity, and accession ID of the top 20 archaeal ASVs in the surface, middle, and deep sediments from the Zhanjiang mangrove wetlands.
**Table S2:** Topological properties of the archaeal co‐occurrence network in the upper sediments (0–15 cm), middle sediments (15–45 cm), and deep sediments (45–90 cm).
**Table S3:** Keystones identified in the archaeal co‐occurrence networks from the upper sediments (0–15 cm), middle sediments (15–45 cm), and deep sediments (45–90 cm).

## Data Availability

All raw 16S rRNA gene sequence datasets generated in this study have been submitted to the NCBI Sequence Read Archive (SRA) database under the accession number PRJNA1199774 and PRJNA1202656.
